# Supplementary material for: Identification of photoperiod-induced specific miRNAs in the adrenal glands of Sunite sheep (Ovis aries)
Source: Front Vet Sci. 2022 Jul 22;9:888207. doi: 10.3389/fvets.2022.888207 (PMC9354845; doi:10.3389/fvets.2022.888207)
Supplement: Supplementary file 1 [file Data_Sheet_1.ZIP › Supplementary file/miRDeep2 file.zip!/miRDeep2 file/Novel_Structure/SP42A3/Novel_313.pdf]

Provisional ID : NC_019460.2_7134
Score total : 328.4
Score for star read(s) : 3.9
Score for read counts : 320.8
Score for mfe : 2.1
Score for randfold : 1.6
Score for cons. seed :
Total read count : 641
Mature read count : 366
Loop read count : 0
Star read count : 275

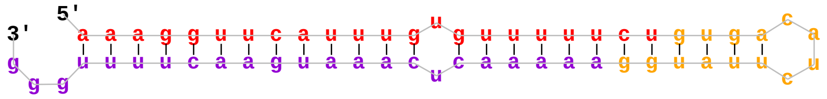

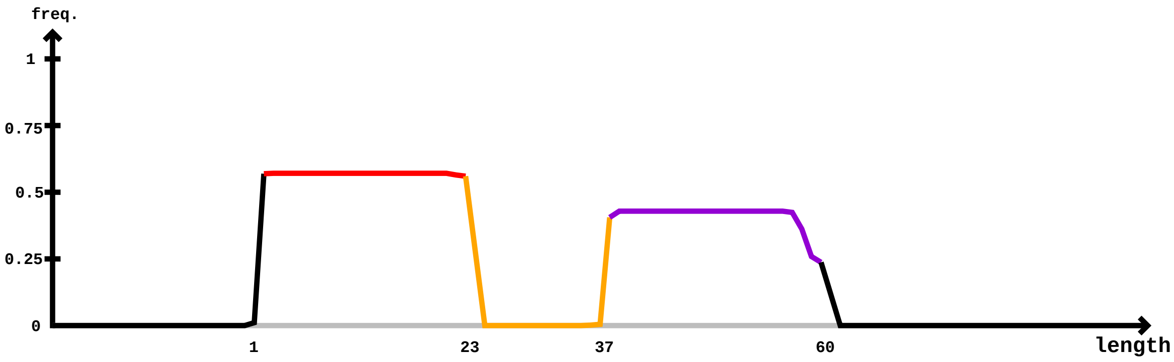


```
                    Mature                                      Star
5'- aaauaauacuggguuggccaaaagguucauuuguguuuuucugugacaucuuauggaaaaacucaaaugaacuuuugggccaacccaaugaaaagcuccaaucccuucacuc   -3'    obs
    aaauaauacuggguuggccaaaagguucauuuguguuuuucugugacaucuuauggaaaaacucaaaugaacuuuuggGccaacccaaugaaaagcuccaaucccuucacuc          exp
    .........(((((((((.((((((((((((.(((((((((((....))))))))))).)))))))))))).))))))))).............................     reads   mm      sample
    ...................aaaagguucauuugGguuuuu........................................................................     2       1       seq
    ...................aaaagguucauuuguguuuuu........................................................................     2       0       seq
    ...................aaaagguucauuuguguuuuuc.......................................................................     3       0       seq
    ....................aaagguucauuuguguuuuucu......................................................................     357     0       seq
    ....................aaagguucauuuguguuuuucug.....................................................................     1       0       seq
    .....................aagguucauuuguguuuuucu......................................................................     1       0       seq
    ......................................................ggaaaaacucaaaugaacuuuuU...................................     1       1       seq
    .......................................................gaaaaacucaaaugaacuuuug...................................     2       0       seq
    ........................................................aaaaacucaaaugaacuuu.....................................     1       0       seq
    ........................................................aaaaacCcaaaugaacuuu.....................................     1       1       seq
    ........................................................aaaaacucaaaugaacuuuu....................................     34      0       seq
    ........................................................aaaaacucaaaugaacuuuA....................................     1       1       seq
    ........................................................aaaaacucaaaugaacuuuG....................................     4       1       seq
    ........................................................aaaaacucaGaugaacuuuug...................................     2       1       seq
    ........................................................aaaaacucaaaugaacuuuuA...................................     52      1       seq
    ........................................................aaaaacCcaaaugaacuuuug...................................     2       1       seq
    ........................................................aaaaacucaaaugaacuuuuU...................................     3       1       seq
    ........................................................aaaaGcucaaaugaacuuuug...................................     1       1       seq
    ........................................................Gaaaacucaaaugaacuuuug...................................     1       1       seq
    ........................................................aaaaacucaaaCgaacuuuugg..................................     2       1       seq
    ........................................................aaaaacCcaaaugaacuuuugg..................................     1       1       seq
    ........................................................aaaaacucaaaugaacuuuuUg..................................     6       1       seq
    ........................................................aaaaacucaGaugaacuuuugg..................................     1       1       seq
    ........................................................aaaaacucaaaugaacuuuuggU.................................     144     1       seq
    ........................................................aaaaacucaaaugaacuuuuUgg.................................     1       1       seq
    .........................................................aaaacCcaaaugaacuuu.....................................     1       1       seq
    .........................................................aaaacucaaaugaacuuuu....................................     1       0       seq
    .........................................................aaaacucaaaugaacuuuug...................................     2       0       seq
    .........................................................aaaacucaaaugaacuuuuUg..................................     4       1       seq
    .........................................................aaaacucaaaugaacuuuuggU.................................     6       1       seq
    .........................................................aaaacucaaaugaacuuuuggg.................................     1       0       seq
```
